# Use of Cardiopulmonary Bypass for Complex Robotic Left Upper Lobectomy After Prior Mantle Irradiation

**DOI:** 10.1016/j.atssr.2025.10.008

**Published:** 2025-11-13

**Authors:** Constantine M. Poulos, Ryan Cassidy, Lana Schumacher

**Affiliations:** 1 Division of Oncology, Danbury Hospital–Nuvance Health/Northwell Health, Danbury, Connecticut; 2 Division of Thoracic Surgery, Tufts Medical Center, Boston, Massachusetts

## Abstract

We discuss the case of a 58-year-old woman with a history of mantle irradiation who presented later in life with a 3.7-cm left upper lobe squamous cell carcinoma. Because of the complexity of the case, all the necessary preoperative precautions were taken in case cardiopulmonary bypass (CPB) became necessary. The patient underwent a completely robotic left upper lobectomy under peripheral CPB. Here we highlight the utility of CPB to enable minimally invasive robotic resections of large, high-risk tumors.

Because of the risks associated with cardiopulmonary bypass (CPB), it is not often viewed as a common adjunct for lung cancer resections. However, with complex lung surgery, CPB can be a useful adjunct to enable the safe completion of minimally invasive resections. Increasingly, surgical candidates are at higher risk because of previous surgery, neoadjuvant therapies, or prior irradiation. In these cases, peripheral partial CPB can increase surgical safety and reduce the risk of intraoperative complications.

This case report is of a 58-year-old woman with a newly diagnosed 3.7-cm left upper lobe mass abutting the pericardium (Video). She initially presented with complaints of acute-onset chest pain, cough, fatigue, and exertional dyspnea. Initial computed tomography showed an isolated 3.7-cm left upper lobe mass abutting the pericardium and mediastinum. Subsequent positron emission tomography showed significant fluorodeoxyglucose avidity without any associated hypermetabolic lymphadenopathy. Robotic navigational bronchoscopy was consistent with keratinizing squamous cell carcinoma. Invasive mediastinal staging and brain magnetic resonance imaging findings were also normal. Given the evidence of elevated hemidiaphragm on initial computed tomography and presumed phrenic nerve involvement with diaphragm paralysis, this tumor was preoperatively staged as cT3 N0 M0. Pulmonary function testing showed a forced expiratory volume in 1 second of 58% and a diffusion capacity of the lungs for carbon monoxide of 51% and thus permissive for high-risk lobectomy.

The patient’s medical history was significant for Hodgkin lymphoma at age 15 years treated with mantle field irradiation. The initial mantle field irradiation was dosed at 36 Gy, but the patient required additional focused doses of 20 Gy to the lower border of C7 and 16 Gy to the left upper lobe because of bulky disease. An additional 19 Gy was later given to the mediastinum because of local recurrence. In addition to this newly diagnosed non-small cell lung cancer (NSCLC), additional sequelae of this intense radiation therapy included left shoulder osteoarthropathy requiring shoulder replacement and left parotid squamous cell carcinoma requiring resection. In addition, preoperative echocardiography showed evidence of radiation-induced cardiomyopathy with reduced left ventricular ejection fraction of 40% and mild to moderate pulmonary hypertension.

After a multidisciplinary tumor board discussion as well as input from the patient, the decision was made to proceed with upfront surgery without induction therapy. Given the high-risk nature of the case in the setting of a large inflammatory mass, prior radiation therapy, and pulmonary hypertension, several precautions were taken. First, a cardiac surgeon, cardiac anesthesiologist, and cardiac perfusionist with a primed CPB circuit were aware and on standby. Preoperative groin access was obtained in the left femoral artery and vein with 8F sheaths. A dedicated vascular tray was open on the field and contained niche instrumentation like laparoscopic bulldogs and video-assisted thoracic surgery vascular clamps necessary in case of bleeding.

A standard robotic left lobectomy approach was used except that the most anterior port was created as a 3-cm incision with a small gel port that can accommodate the introduction of bulldog clamps, vascular clamps, and sponge sticks for emergencies. Immediately on entering the chest, there was a large, densely fibrotic mass involving the left anterior upper lobe, mediastinum, and pericardium. Given the patient’s prior radiation therapy, the posterior mediastinal plane and fissure were largely inaccessible. In addition, because of the dense mediastinal and hilar fibrosis, it was decided to enter within the pericardium in an attempt to gain intrapericardial control of the left main pulmonary artery. Furthermore, the left main pulmonary artery was enlarged, consistent with known pulmonary hypertension, and there were dense adhesions on the posterior aspect of the artery precluding safe dissection.

We began working in the anterior hilum, where the superior pulmonary vein was isolated and divided and the inferior pulmonary vein was encircled, snared by a vessel loop, and secured with a robotic clip applier for further vascular control. The lingular bronchus was sharply divided next in an attempt to identify the anterior pulmonary arterial anatomy. Despite these maneuvers, we did not think that we could safely progress because of the large, obscuring right main pulmonary artery and decided to initiate CPB by upsizing the preplaced 8F left femoral sheaths. The intention was to use peripheral CPB to allow decompression of the pulmonary vasculature to enable a safe, bloodless dissection.

The patient was fully heparinized. By use of the previously placed 8F left femoral sheaths, femoral access was upsized to 17F bypass cannulas through percutaneous Seldinger method. This cannula size was chosen to allow adequate pulmonary vascular decompression and maintenance of systemic perfusion at a cardiac index of 2.0 L·min^−1^·m^−2^ while minimizing the risk of peripheral limb ischemia. Through the anterior gel port, a laparoscopic bulldog clamp was passed into the field and placed across the left main pulmonary artery. With total vascular control of the left lung, the dissection was continued along the superior hilum and left main pulmonary artery until the left upper lobe branches were adequately identified. These were serially transected with a white-loaded robotic stapler. A stay stitch was used to retract the transected lingular bronchus, and the upper division bronchus was divided with a single fire of a green-loaded robotic stapler. Last, once all the relevant structures had been divided, the fissure was completed with serial fires of a robotic stapler. A thorough lymphadenectomy was performed and the specimens were removed.

The patient was weaned from CPB and the chest was closed. Protamine was given, and the patient was repositioned supine to facilitate groin cutdown for removal of peripheral bypass cannulas.

The patient was monitored postoperatively in the cardiothoracic intensive care unit. She was treated for atrial fibrillation with rapid ventricular response and ultimately had 1 episode of symptomatic bradycardia that resolved without intervention. Final pathologic examination showed pT3 N0 M0 keratinizing squamous cell carcinoma. The patient was offered adjuvant chemotherapy but completed only 1 cycle because of intolerance. She is currently maintained on immunotherapy.

## Comment

Patients who received mantle field irradiation during adolescence are now reaching ages at which secondary malignant neoplasms are emerging.^1^ This presents a significant challenge for thoracic surgeons as prior radiation therapy induces dense inflammatory changes that can complicate surgical approaches for secondary lung cancers. Whereas other maneuvers, like converting to open or the initiation of extracorporeal membrane oxygenation, are useful tools for complex cases, CPB allows pulmonary vascular decompression and a relatively bloodless field to facilitate safe dissection while maintaining systemic perfusion. Other high-risk cases in which adjunct CPB may be necessary include include tumors with invasion of the main pulmonary artery, intense fibrotic reactions from previously neoadjuvant therapies, large nodal involvement of critical structures, and patients with pulmonary hypertension.

Studies have reported that CPB can be a safe and effective adjunct in lung cancer surgery.^2^^,^^3^ Langer and coworkers,^2^ in a series of 20 patients who underwent resection of T4 NSCLC with CPB assistance, found no significant difference in overall or disease-free survival compared with patients who underwent resection without CPB. Notably, the CPB group had a higher rate of R0 resection.

A systematic review by Muralidaran and coworkers^3^ examining long-term outcomes in patients undergoing CPB-assisted NSCLC resection found significantly improved survival when planned preoperatively. In the presented case, we engaged our cardiac surgery and anesthesia colleagues early to enable an “all-hands-on-deck” situation if necessary. Lower extremity vascular access was obtained before lateral positioning in case emergent bypass became necessary. Last, having a tray of vascular robotic instruments open and readily available eliminates the potential for error in case of vascular catastrophe.

Currently, no literature exists on the use of partial CPB to facilitate robotic NSCLC resection. This case underscores the potential role of CPB as a valuable adjunct for thoracic surgeons performing complex, high-risk, minimally invasive procedures. Particularly in patients with pulmonary hypertension, partial CPB may enhance surgical safety and feasibility. Successful implementation requires meticulous preoperative planning and close coordination with cardiac surgery, cardiac anesthesia, and perfusion teams to ensure optimal patient outcomes.

## References

[1] Dracham C.B., Shankar A., Madan R. (2018). Radiation induced secondary malignancies: a review article. Radiat Oncol J.

[2] Langer N.B., Mercier O., Fabre D. (2016). Outcomes after resection of T4 non-small cell lung cancer using cardiopulmonary bypass. Ann Thorac Surg.

[3] Muralidaran A., Detterbeck F.C., Boffa D.J., Wang Z., Kim A.W. (2011). Long-term survival after lung resection for non–small cell lung cancer with circulatory bypass: a systematic review. J Thorac Cardiovasc Surg.

